# Heterogeneous effects of cytotoxic chemotherapies for platinum-resistant ovarian cancer

**DOI:** 10.1007/s10147-023-02367-1

**Published:** 2023-06-22

**Authors:** Katsuhiko Nara, Ayumi Taguchi, Takehito Yamamoto, Konan Hara, Yuri Tojima, Harunori Honjoh, Akira Nishijima, Satoko Eguchi, Yuichiro Miyamoto, Kenbun Sone, Mayuyo Mori, Tappei Takada, Yutaka Osuga

**Affiliations:** 1 Department of Pharmacy, The University of Tokyo Hospital, Tokyo, Japan; 2 Department of Obstetrics and Gynecology, The University of Tokyo, 7-3-1 Hongo, Bunkyo-ku, Tokyo, 113-8655 Japan; 3 Laboratory of Human Single Cell Immunology, World Premier International Immunology Frontier Research Center (WPI-IFReC), Osaka University, Suita, Japan; 4 The Education Center for Clinical Pharmacy, Graduate School of Pharmaceutical Sciences, The University of Tokyo, Tokyo, Japan; 5 Department of Economics, The University of Arizona, Tucson, USA

**Keywords:** Platinum-resistant ovarian cancer, Heterogeneous treatment effect, Adverse event, Histology, Platinum-free interval

## Abstract

**Background:**

Single-agent chemotherapy with or without bevacizumab (Bev) is a standard therapy for platinum-resistant ovarian cancer (PR-OC). However, there is a lack of literature on chemotherapy agent selection in heterogenous PR-OC. Therefore, we aimed to clarify the heterogeneous treatment effects of each chemotherapy agent.

**Methods:**

Patients who underwent single-drug chemotherapy agents or Bev combination therapy for PR-OC between January 2009 and June 2022 were included in this study. We assessed the impact of each chemotherapy agent on the time to treatment failure (TTF) according to histological type, platinum-free interval (PFI), and Bev usage.

**Results:**

A total of 158 patients received 343 different chemotherapy regimens. In patients with clear cell carcinoma/mucinous carcinoma (CC/MC), gemcitabine (GEM) had the strongest effect with a median TTF of 5.3 months, whilst nedaplatin (NDP) had the lowest effect with a median TTF of 1.4 months. In contrast, in the non-CC/MC group, irinotecan (CPT-11) and NDP had a better TTF than GEM and pegylated liposomal doxorubicin (PLD). There were notable differences in the treatment efficacy of NDP according to PFI. Specifically, NDP prolonged the TTF in patients with a PFI ≥ 3 months. Compared with GEM alone, GEM + Bev tended to prolong the TTF more effectively; however, an additive effect was not observed with PLD + Bev.

**Conclusions:**

This study demonstrated that the effect of chemotherapy agents differed according to the tumor and background characteristics of the patient. Our findings will improve selection of effective therapies for patients with PR-OC by considering their background characteristics.

**Supplementary Information:**

The online version contains supplementary material available at 10.1007/s10147-023-02367-1.

## Introduction

Worldwide, ovarian cancer (OC) is the eighth most common cancer and the eighth most common cause of cancer-related death in women [1]. The OC morbidity rate is increasing in Japan and its age-adjusted mortality rate is increasing worldwide (3.2 in 10,000 people) [2].

The standard therapies for OC are surgery and platinum-based chemotherapy [3, 4]. For platinum-sensitive recurrent OC (platinum-free interval [PFI] ≥ 6 months [Mos]), the standard protocol involves re-administering platinum combination chemotherapy given the expected response to platinum agents. Contrarily, a single-drug chemotherapy agent is recommended for platinum-resistant OC (PR-OC; PFI ≤ 6 Mos), considering its efficacy and adverse events (AEs) [3, 4]. However, single-drug chemotherapy agents often have an insufficient therapeutic effect, with a response rate of 10–20% and progression-free survival (PFS) of 3–4 Mos [5–9]. Chemotherapy agents combined with bevacizumab (Bev) therapy significantly prolong PFS in patients with PR-OC and are increasingly used for patients without contraindications to concomitant Bev therapy [8, 10].

Chemotherapy sensitivity in PR-OC varies by histologic type. Serous carcinoma (SC) and endometrioid carcinoma (EMC) are notably sensitive, while clear cell carcinoma (CC) and mucinous carcinoma (MC) exhibit lower sensitivity [11–13]. Factors influencing chemotherapy effectiveness include the choice of treatment lines, combination with Bev, PFI, ascites presence, cancer antigen (CA) levels 125 levels, age, and performance status [10, 14–18]. Therefore, determining PR-OC treatments should consider the patient’s background and tumor characteristics. Nonetheless, the heterogeneous effects of each drug on PR-OC remain unclear.

Quality of life (QOL) should be considered when determining therapeutic strategies for PR-OC since patients with recurrent OC have decreased physical fitness and bone marrow reserves after undergoing multiple treatment regimens. Moreover, there are several life-threatening AEs related to chemotherapy agents, including thromboembolic events and interstitial pneumonia (IP); therefore, it is important to elucidate the incidence and timing of their occurrence.

This retrospective study aimed to examine the therapeutic effects and AEs in patients with PR-OC who underwent single-drug chemotherapy agent or Bev combination therapy according to the patient’s background and tumor characteristics.

## Materials and methods

### Patients and chemotherapy

We included patients with PR-OC from The University of Tokyo Hospital who were treated with a single chemotherapy agent (irinotecan [CPT-11], pegylated liposomal doxorubicin [PLD], gemcitabine [GEM], paclitaxel [PTX], nogitecan [NGT], nedaplatin [NDP], or docetaxel [Doc]) or Bev combination therapy between January 2009 and June 2022. All single chemotherapy agents were administered intravenously, CPT-11 at a dose of 100 mg/m^2^ over 90 min on Days 1, 8, and 15 per cycle, PLD at a dose of 50 mg/m^2^ over 90 min on Day 1 per cycle, GEM at a dose of 1000 mg/m^2^ for 30 min on Days 1, 8, and 15 per cycle, PTX at a dose of 80 mg/m^2^ over 60 min on Days 1, 8, and 15 per cycle; NGT at a dose of 1.25 mg/m^2^ for 30 min from Day 1 to Day 5 per cycle, NDP at a dose of 80 mg/m^2^ over 60 min on Day 1 per cycle, and Doc at a dose of 70 mg/m^2^ over 60 min on Day 1 per cycle. Bev combination therapy comprised adding Bev 15 mg/kg on Day 1 to the dosing schedule of PLD, PTX, and NGT. GEM + Bev therapy comprised intravenously administration of GEM at a dose of 1000 mg/m^2^ for 30 min on Days 1 and 8 per cycle and Bev 15 mg/kg on Day 1. Bev was administered over 90, 60, and 30 min for the first, second, and third (and subsequent) times, respectively. Cycle duration was 28 days for all drugs, except Doc and GEM + Bev (both 21 days). The initial dose of chemotherapy agents was reduced in accordance with the patient's condition. The chemotherapy agents order and the inclusion of Bev depend on the patient’s prior drug history, complications, and general condition. The exclusion criteria were pathological diagnosis of squamous cell carcinoma or neuroendocrine tumor and death from causes other than the primary disease within a week after chemotherapy initiation.

### Data collection and clinical outcomes

We obtained the following data from the electronic medical records: the International Federation of Gynecology and Obstetrics (FIGO) stage at diagnosis, treatment history of platinum-based chemotherapy, PFI, time to treatment failure (TTF), treatment line after platinum resistance (first line, second line, or more), histology (CC/MC or non-CC/MC), background characteristics before starting chemotherapy for platinum resistance (age, body mass index), ascites status, use of Bev, initial dose, and AEs. The primary study endpoint was TTF because the decision to continue treatment is often based not only on disease progression but also on a comprehensive assessment of AEs and QOL. TTF was defined as the interval from the date of chemotherapy initiation to death due to primary disease, primary disease progression (disease progression-associated bowel obstruction), or treatment discontinuation due to AEs, whichever occurred first. AEs were assessed based on the Common Terminology Criteria for Adverse Events version 5.0 and were assessed at intervals between the start and end of each chemotherapy regimen. Platinum resistance was defined as recurrence occurring ≤ 6 Mos after completion of platinum-based chemotherapy. PFI was defined as the interval from the date of the last platinum-based chemotherapy dose to the date of first diagnosis of platinum-resistant recurrence.

We evaluated the presence/absence of ascites, and the use of Bev before the administration of each chemotherapy agent.

### Statistical analyses

The Kaplan–Meier method was used to analyze TTF. The log-rank test was used to analyze differences in TTF according to chemotherapy agent, histology, PFI, usage of Bev, and treatment line. Differences in TTF among CPT-11, GEM, PLD, and NDP in the CC/MC, non-CC/MC, PFI ≥ 3 Mos, and PFI < 3 Mos groups were evaluated by the log-rank test. Differences in TTF between GEM and GEM + Bev and between PLD and PLD + Bev were also analyzed using the log-rank test. Using the univariate Cox proportional hazards model, we estimated the hazard ratio (HR) for TTF events according to chemotherapy agent between each factor including histology, PFI, and Bev usage. The incidence rate of each Grade ≥ 3 AE, including neutropenia, thrombocytopenia, anemia, febrile neutropenia, anorexia, nausea, diarrhea, aspartate transaminase (AST)/alanine transaminase (ALT) increase, hand-foot syndrome (HFS), rash, fatigue, oral mucositis, IP, and thromboembolic events, was calculated according to chemotherapy agent. All tests were two-tailed. Statistical significance was set at *P* < 0.05. All statistical analyses were performed using SPSS Statistics for Windows version 24 (IBM Corp., Armonk, NY).

### Ethics approval

The study protocol was approved by the ethics committee of the Faculty of Medicine, University of Tokyo (Approval number: 2654, 3084, 209127NI). The institutional review board granted an opt-out recruitment approach and waived the need for written informed consent. This study adhered to the Declaration of Helsinki.

## Results

### Patient background

Among 161 patients who received chemotherapy for PR-OC, we excluded three patients (two patients without adenocarcinoma and one patient who died of aspiration pneumonia the day following chemotherapy). A total of 158 patients received 343 different chemotherapy regimens. The median age of the patients was 61.1 years and > 70% of patients had FIGO stage ≥ 3 at diagnosis (Table 1). Approximately 50% of the patients had SC and 25% had CC. CPT-11 was the most commonly administered drug, followed by GEM and PLD. Table 2 summarizes the usage characteristics of each chemotherapy agent. Approximately 80% of patients who received CPT-11 received it as first-line treatment, while 70% of patients who received NDP and PTX received it as third- or fourth-line treatment. During the PTX administration, most patients had a history of combined PTX and platinum treatment. Conversely, a small number had a history of other chemotherapy regimens.

**Table 1:** Characteristics of patients before the first-line chemotherapy

Characteristics	All patients (*n* = 158)
Age [year], median (range)	61.1 (36.4–84.5)
Age ≥ 65 years, *n* (%)	65 (41.1)
BMI [kg/m^2^], median (range)	21.5 (12.2–36.2)
FIGO stage (≥ 3), *n* (%)	125 (79.1)
Primary site, ovarian (%)	144 (91.1)
Histology, *n* (%)	
Serous	79 (50.0)
Clear cell	41 (25.9)
Endometrioid	16 (10.1)
Mucinous	5 (3.2)
Others	17 (10.8)
Ascites, *n* (%)	68 (43.0)
History of platinum-based chemotherapy plus bevacizumab, *n* (%)	41 (25.9)
Platinum-free interval < 3 months, *n* (%)	100 (63.3)
Platinum-resistant cytotoxic chemotherapy, *n* (%)	
Irinotecan	92 (58.2)
Gemcitabine	90 (57.0)
Pegylated liposomal doxorubicin	82 (51.9)
Nedaplatin	33 (20.9)
Paclitaxel	18 (11.4)
Nogitecan	14 (8.9)
Docetaxel	14 (8.9)
Bevacizumab combination therapy, *n* (%)	49 (31.0)

*BMI* body mass index, *FIGO* International Federation of Gynecology and Obstetrics

**Table 2:** Background of platinum-resistant chemotherapy agents

Characteristics	CPT-11	GEM	PLD	NDP	PTX	NGT	Doc
	(*n* = 92)	(*n* = 90)	(*n* = 82)	(*n* = 33)	(*n* = 18)	(*n* = 14)	(*n* = 14)
Treatment line after platinum resistance							
1st line	70 (76.1)	36 (40.0)	40 (48.8)	0 (0.0)	3 (16.7)	1 (7.1)	7 (50.0)
2nd line	21 (22.8)	33 (36.7)	22 (26.8)	10 (30.3)	2 (11.1)	5 (35.7)	3 (21.4)
3rd line	1 (1.1)	16 (17.8)	14 (17.1)	15 (45.5)	7 (38.9)	1 (7.1)	2 (14.3)
4th line	0 (0.0)	5 (5.6)	6 (7.3)	8 (24.2)	6 (33.3)	7 (50.0)	2 (14.3)
Bevacizumab therapy	0 (0.0)	28 (31.1)	20 (24.4)	0 (0.0)	16 (88.9)	2 (14.3)	0 (0.0)
Treatment line of bevacizumab therapy							
1st line	0 (0.0)	18 (20.0)	13 (15.9)	0 (0.0)	3 (16.7)	1 (7.1)	0 (0.0)
2nd line	0 (0.0)	8 (8.9)	3 (3.7)	0 (0.0)	2 (11.1)	1 (7.1)	0 (0.0)
3rd line or more	0 (0.0)	2 (2.2)	4 (4.9)	0 (0.0)	11 (61.1)	0 (0.0)	0 (0.0)
Initial dose, full dose	45 (48.9)	36 (40.0)	39 (47.6)	31 (93.9)	16 (88.9)	12 (85.7)	12 (85.7)
Platinum-free interval < 3 months, *n* (%)	58 (63.0)	55 (61.1)	50 (61.0)	18 (54.5)	8 (44.4)	6 (42.9)	9 (64.3)
Histology							
Serous	40 (43.5)	45 (50.0)	50 (61.0)	20 (60.6)	14 (77.8)	10 (71.4)	7 (50.0)
Endometrioid	11 (12.0)	8 (8.9)	10 (12.2)	2 (6.1)	2 (11.1)	0 (0.0)	2 (14.3)
Clear cell	26 (28.3)	24 (26.7)	11 (13.4)	7 (21.2)	1 (5.6)	2 (14.3)	3 (21.4)
Mucinous	2 (2.2)	1 (1.1)	4 (4.9)	0 (0.0)	0 (0.0)	0 (0.0)	1 (7.1)
Others	13 (14.1)	12 (13.3)	7 (8.5)	4 (12.1)	1 (5.6)	2 (14.3)	1 (7.1)
Treatment history of the same drug in platinum-containing regimens	0 (0.0)	1 (1.1)	0 (0.0)	1 (3.0)	17 (94.4)	0 (0.0)	1 (7.1)
Ascites	37 (40.2)	37 (41.1)	32 (39.0)	12 (36.4)	9 (50.0)	6 (42.9)	4 (28.6)

Data are shown as *n* (%). *CPT-11* irinotecan, *PLD* pegylated liposomal doxorubicin, *GEM* gemcitabine, *PTX* paclitaxel, *NGT* nogitecan, *NDP* nedaplatin, *Doc* docetaxel

### Comparison of TTF according to chemotherapy agent, background, and tumor characteristics

**Fig. 1:**
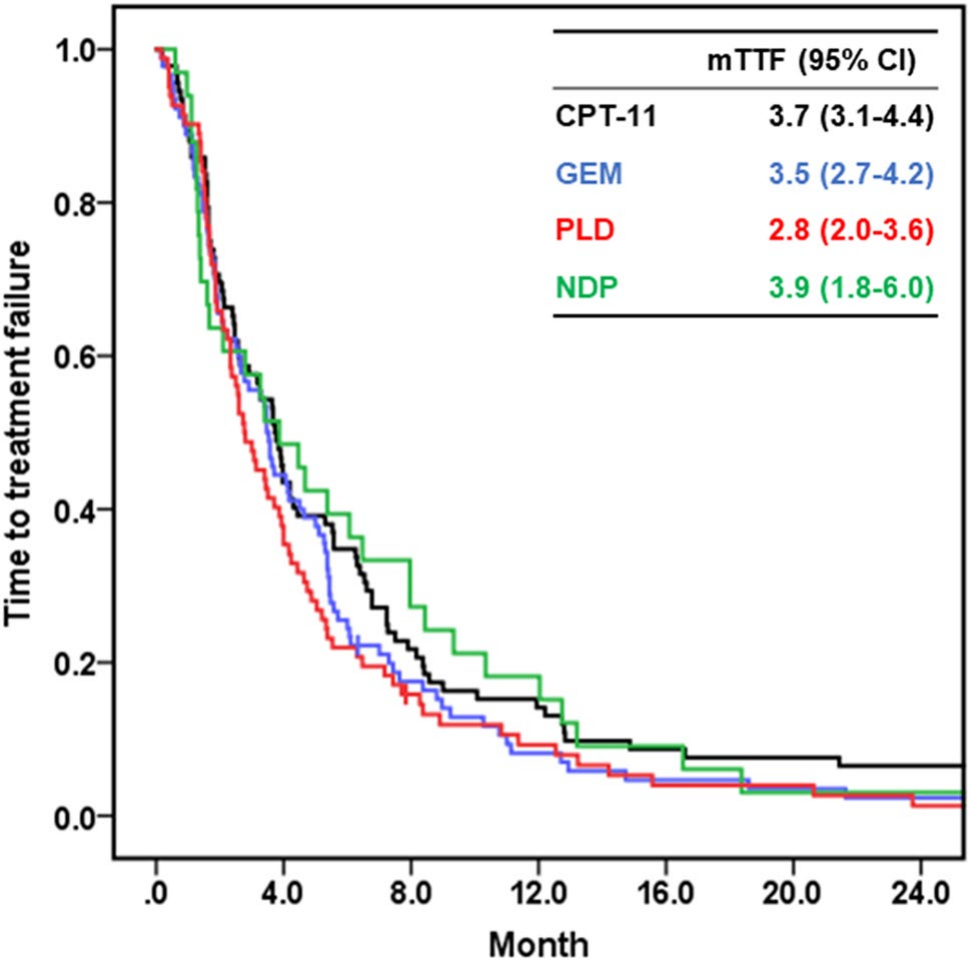
Kaplan–Meier estimates of the time to treatment failure according to the type of chemotherapy agent. TTF according to the type of chemotherapy. The differences in TTF between each factor were evaluated using the log-rank test. *TTF* time to treatment failure, *CPT-11* irinotecan, *GEM* gemcitabine, *PLD* pegylated liposomal doxorubicin, *NDP* nedaplatin, *mTTF* median time to treatment failure, *CI* confidence interval

In all regimens, approximately 90% of patients discontinued chemotherapy agents due to disease progression (Table 3). TTF was compared according to chemotherapy agent, background characteristics, and tumor characteristics. The comparison of TTF by chemotherapy agent was performed with four frequently used chemotherapy agents: CPT-11, GEM, PLD, and NDP. The median TTF of each drug ranged from 2–4 Mos, with no significant differences (Fig. 1). There were no significant differences in TTF among the histological types (Supplementary Fig. 1a), between PFI ≥ 3 Mos and < 3 Mos (Supplementary Fig. 1b), or between first-line treatment and second- or later-line treatments (Supplementary Fig. 1c). Patients who used Bev or those without ascites had a better TTF than those who did not use Bev or those with ascites (Supplementary Fig. 1d, e).

**Table 3:** Reasons for discontinuation or change of chemotherapy agents

	CPT-11	GEM	PLD	NDP	PTX	NGT	Doc
	(*n* = 92)	(*n* = 90)	(*n* = 82)	(*n* = 33)	(*n* = 18)	(*n* = 14)	(*n* = 14)
Progression disease	87 (94.6)	81 (90.0)	69 (84.1)	32 (97.0)	16 (88.9)	13 (92.9)	13 (92.9)
Adverse events	3 (3.3)	9 (10.0)	9 (11.0)	1 (3.0)	1 (5.6)	1 (7.1)	1 (7.1)
Patient’s hope	0 (0.0)	0 (0.0)	1 (1.2)	0 (0.0)	1 (5.6)	0 (0.0)	0 (0.0)
Others	2 (2.2)	0 (0.0)	3 (3.7)	0 (0.0)	0 (0.0)	0 (0.0)	0 (0.0)

Data are shown as *n* (%). *CPT-11* irinotecan, *GEM* gemcitabine, *PLD* pegylated liposomal doxorubicin, *NDP* nedaplatin, *PTX* paclitaxel, *NGT* nogitecan, *Doc* docetaxel

#### Histology

**Fig. 2:**
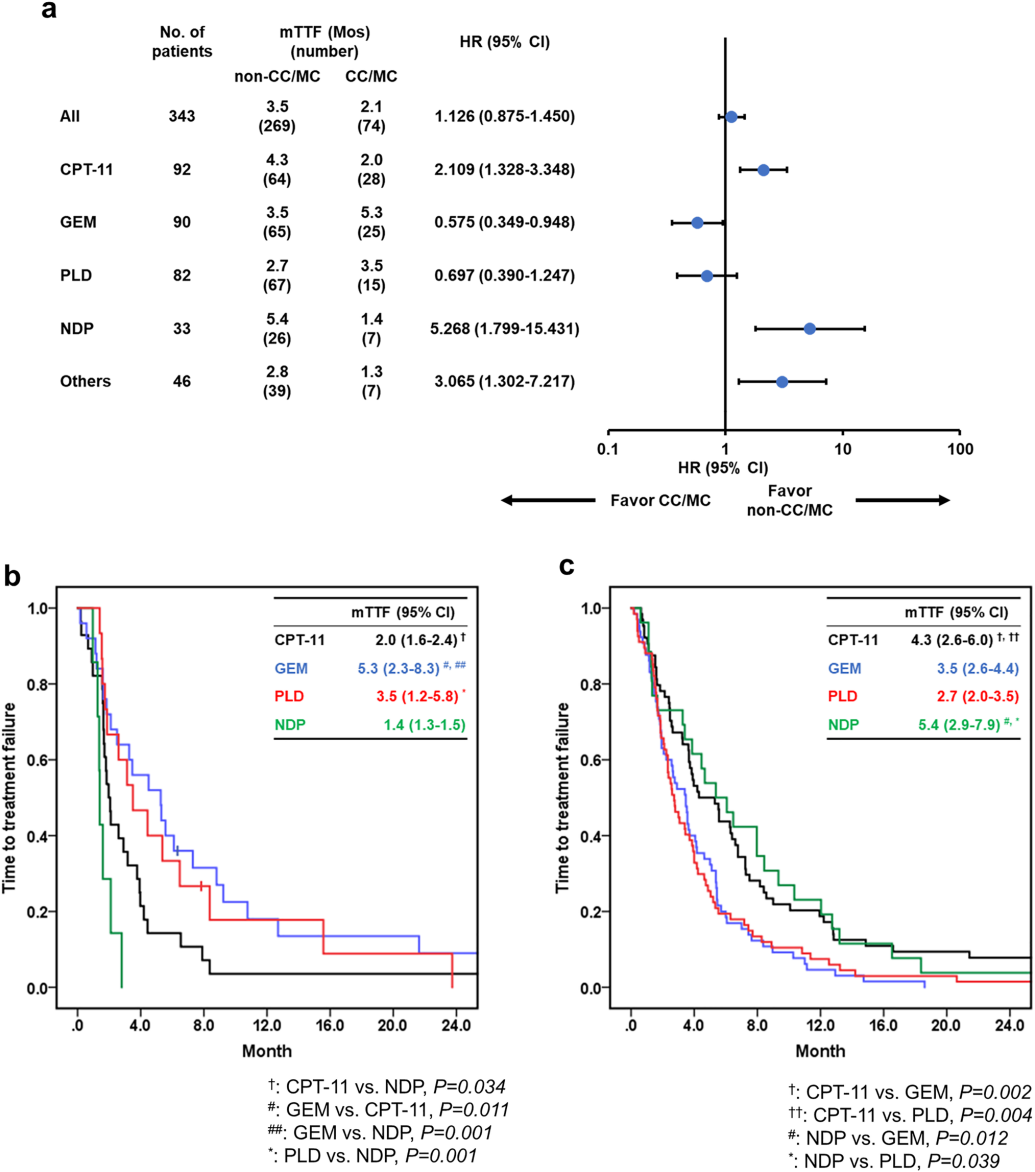
Forest plot and Kaplan–Meier estimates of the time to treatment failure according to histology (CC/MC vs. non-CC/MC). The median TTF calculated by the Kaplan–Meier analysis and the HR calculated by the univariate Cox proportional hazard model for each chemotherapy agent are shown in part (**a**). The TTF of each chemotherapy for CC/MC (**b**) and non-CC/MC (**c**) is shown. In parts (**b**) and (**c**), differences in TTF between each factor were evaluated using the log-rank test. *TTF* time to treatment failure, *CC/MC* clear cell carcinoma/mucinous carcinoma, *CPT-11* irinotecan, *GEM* gemcitabine, *PLD* pegylated liposomal doxorubicin, *NDP* nedaplatin, *HR* hazard ratio, *CI* confidence interval, *mTTF*, median time to treatment failure

Figure 2 summarizes the TTF and HR of the chemotherapy agents for each histologic type (CC/MC vs. non-CC/MC). PTX, NGT, and Doc were classified as “others” since they were administered only to a few patients. TTF tended to be worse in the CC/MC group (HR: 1.126, 95% confidence interval [CI]: 0.875–1.450). However, the effectiveness of the chemotherapy agents differed according to the histological type. Specifically, the TTF of CPT-11 and NDP was significantly longer in the non-CC/MC group than in the CC/MC group (HR: 2.109 and 5.268, respectively) (Fig. 2a). Further, the TTF of GEM was significantly longer in the CC/MC group than in the non-CC/MC group (HR: 0.575), and the TTF of PLD tended to be longer in the CC/MC group than in the non-CC/MC group (HR: 0.697). We next compared the efficacy of each chemotherapy agent separately for each histologic type. In the CC/MC, GEM had the strongest effect with a median TTF of 5.3 Mos and NDP had the weakest effect with a median TTF of 1.4 Mos (Fig. 2b). In the non-CC/MC group, CPT-11 and NDP had a better TTF than GEM and PLD (Fig. 2c).

#### Platinum-free interval

**Fig. 3:**
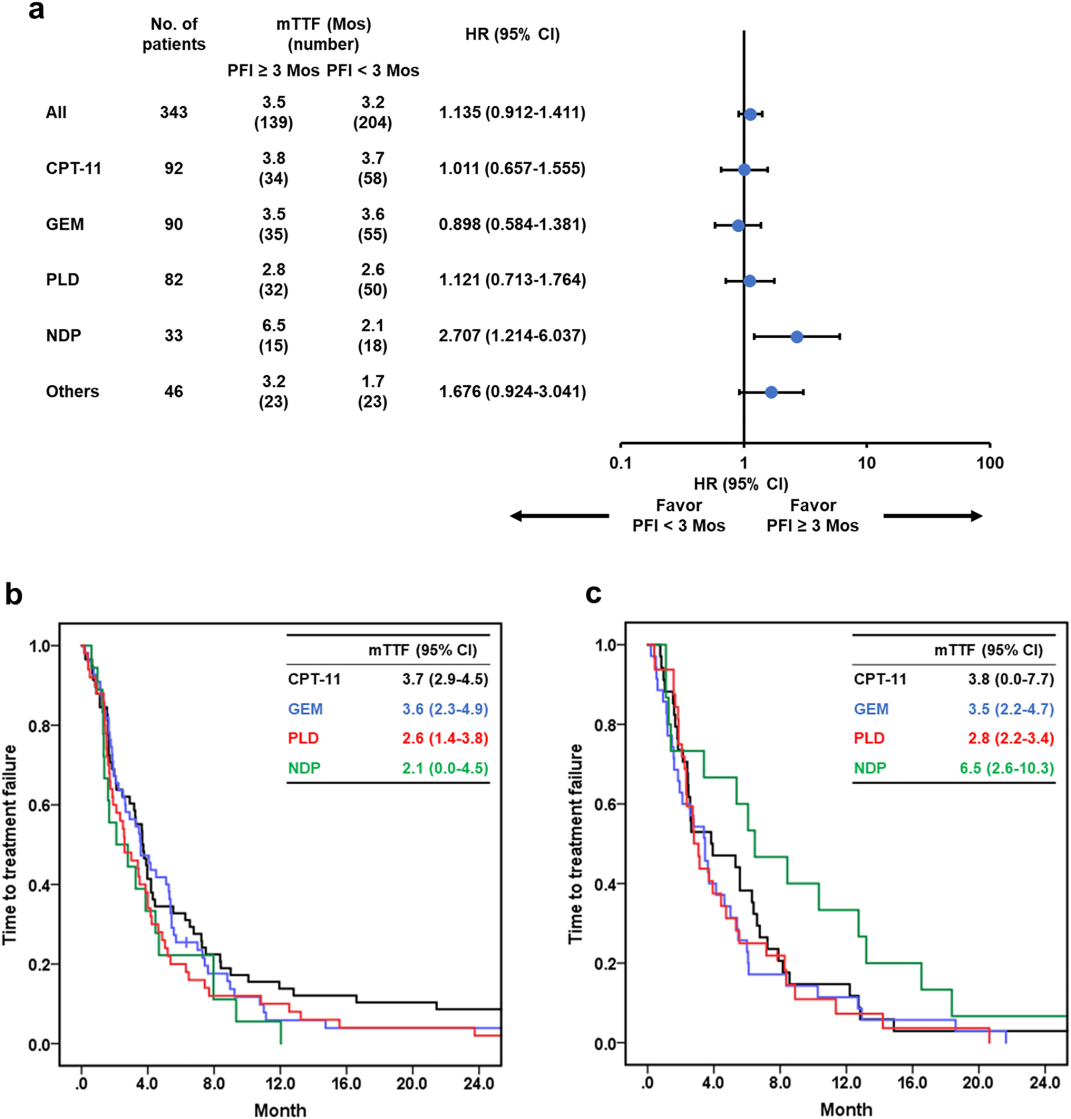
Forest plot and Kaplan–Meier estimates of the time to treatment failure of platinum-free interval (PFI ≥ 3 Mos vs. PFI < 3 Mos). The median TTF calculated by the Kaplan–Meier curve and the HR calculated by the univariate Cox proportional hazard model for each chemotherapy agent are shown in part (**a**). The TTF of each chemotherapy agent for patients with PFI ≥ 3 Mos (**b**) and those with PFI < 3 Mos (**c**) is shown. In parts (**b**) and (**c**), differences in TTF between each factor were evaluated using the log-rank test. *TTF* time to treatment failure, *PFI* platinum-free interval, *Mos* months, *CPT-11* irinotecan, *GEM* gemcitabine, *PLD* pegylated liposomal doxorubicin, *NDP* nedaplatin, *HR* hazard ratio, *CI* confidence interval, *mTTF* median time to treatment failure

Comparison of TTF of the chemotherapy agents according to PFI (< 3 Mos vs. ≥ 3 Mos) (Fig. 3) showed that TTF did not differ according to PFI (HR: 1.135; 95% CI: 0.912–1.411). The TTF of NDP was most strongly influenced by PFI; specifically, TTF was significantly shorter in patients with PFI < 3 Mos (HR: 2.707) (Fig. 3a). Moreover, NDP showed the best TTF in the Kaplan–Meier curve for patients with PFI ≥ 3 Mos; however, TTF did not differ among chemotherapy agents in patients with PFI < 3 Mos (Fig. 3b, c).

#### Bevacizumab

**Fig. 4:**
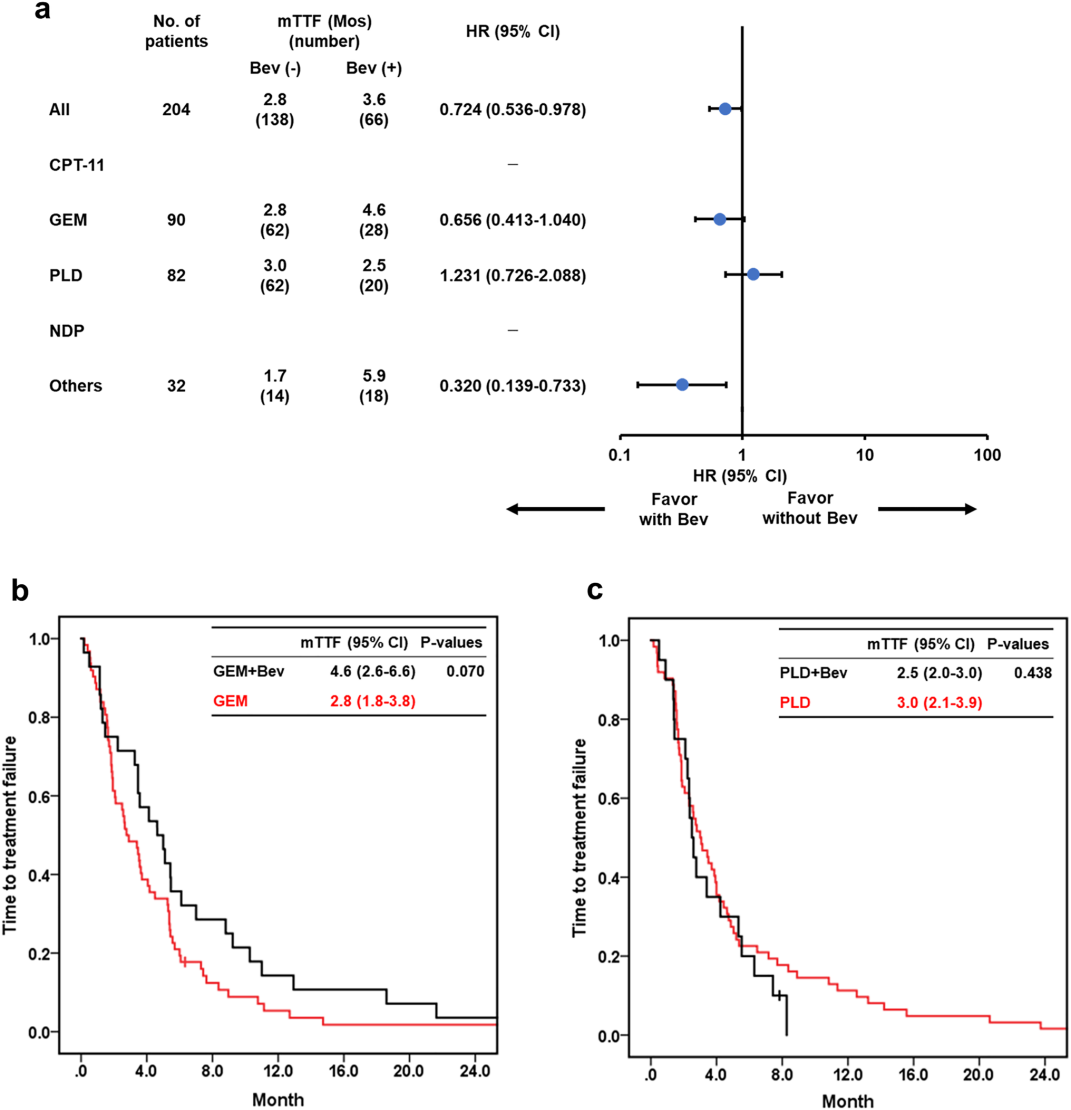
Forest plot and Kaplan–Meier estimates of the time to treatment failure with/without bevacizumab. The median TTF calculated by the Kaplan–Meier curve and HR calculated by the univariate Cox proportional hazard model for each chemotherapy agent are shown in part (**a**). The TTF of GEM/GEM + Bev (**b**) and PLD/PLD + Bev (**c**) is shown. The differences in TTF between each factor were evaluated using the log-rank test. *TTF* time to treatment failure, *Bev* bevacizumab, *CPT-11* irinotecan, *GEM* gemcitabine, *PLD* pegylated liposomal doxorubicin, *NDP* nedaplatin, *HR* hazard ratio, *CI* confidence interval, *mTTF* median time to treatment failure

Comparison of the TTF of each chemotherapy agent according to Bev (Fig. 4) showed that combination therapy with Bev significantly improved TTF (HR: 0.724; 95% CI: 0.536–0.978). However, the improvement effects of Bev on prognosis differed according to the type of chemotherapy agent. Specifically, the Bev combination therapies were most effective in “Others” including PTX and NGT (Fig. 4a), followed by GEM (Fig. 4a, b), while the PLD + Bev combination did not improve TTF (Fig. 4a, c).

#### Presence of ascites

Patients with ascites had a worse TTF, regardless of the type of chemotherapy agent administered (Supplementary Fig. 2).

### Incidence rate and timing of AEs

Table 4 summarizes the Grade ≥ 3 AEs for CPT-11, GEM, PLD, and NDP. The incidence of febrile neutropenia, nausea, and diarrhea was highest with CPT-11 usage, and GEM users showed the highest incidence of neutropenia and AST/ALT elevation (46.5% and 4.7%, respectively). PLD users showed the highest incidence of thromboembolic events, IP, HFS, and oral mucositis. Notably, the incidence of thrombotic events was 10.1%. The main AE associated with NDP was myelosuppression. There were no cases of non-hematologic toxicity in Grade ≥ 3 AEs.

**Table 4:** Adverse events of CPT-11, GEM, PLD, and NDP

Adverse events Grade ≥ 3	CPT-11 (*n* = 92)	GEM (*n* = 90)	PLD (*n* = 82)	NDP (*n* = 33)
Neutropenia	30 (32.6)	41 (45.6)	25 (30.5)	5 (15.2)
Thrombocytopenia	0 (0.0)	5 (5.6)	2 (2.4)	3 (9.1)
Anemia	10 (10.9)	13 (14.4)	12 (14.6)	6 (18.2)
Febrile neutropenia	6 (6.5)	2 (2.2)	1 (1.2)	0 (0.0)
Anorexia	0 (0.0)	0 (0.0)	0 (0.0)	0 (0.0)
Nausea	6 (6.5)	0 (0.0)	0 (0.0)	0 (0.0)
Diarrhea	8 (8.7)	0 (0.0)	0 (0.0)	0 (0.0)
AST/ALT increase	1 (1.1)	4 (4.4)	2 (2.4)	0 (0.0)
Hand-foot syndrome	0 (0.0)	0 (0.0)	3 (3.7)	0 (0.0)
Rash	0 (0.0)	1 (1.1)	0 (0.0)	0 (0.0)
Fatigue	1 (1.1)	0 (0.0)	1 (1.2)	0 (0.0)
Oral mucositis	0 (0.0)	0 (0.0)	3 (3.7)	0 (0.0)
Interstitial pneumonia	0 (0.0)	1 (1.1)	3 (3.7)	0 (0.0)
Thromboembolic event	3 (3.3)	1 (1.1)	8 (9.8)	0 (0.0)

Data are shown as n (%). CPT-11, irinotecan; PLD, pegylated liposomal doxorubicin; GEM, gemcitabine, NDP, nedaplatin; AST, aspartate transaminase; ALT, alanine transaminase

Among the AEs, we investigated the timing of the onset of thromboembolic events and IP for all grades (Supplementary Fig. 3). Although the incidence of both AEs was high during the first- and second- line treatments, thromboembolic events also occurred after the fourth treatment course.

## Discussion

We investigated the TTF of chemotherapy agents and the factors against PR-OC. We found that the impact of histological type, PFI, and Bev usage was dependent on the type of chemotherapy agent. Regarding the AE profiles, PLD showed the highest incidence of thromboembolic events and IP.

There is insufficient evidence for selecting chemotherapy agents for OC based on patient background and tumor characteristics. Accordingly, chemotherapy agents for PR-OC are selected considering treatment history, residual toxicity, cost, convenience, and patient preference [19]. We confirmed that the therapeutic effects of chemotherapy agents differ according to patient background and tumor characteristics.

Each drug’s therapeutic effect differed according to histological type (Fig. 2). Specifically, GEM showed the strongest therapeutic effect in the CC/MC group with TTF > 5 Mos, whilst it showed a poor therapeutic effect in the non-CC/MC group. A preclinical study demonstrated the potential efficacy of GEM for CC [20]. Additionally, a multi-center Italian trial on OC (MITO)-09 confirmed a high response rate of CC to GEM [21]. This suggests that GEM is the preferred therapeutic candidate in CC/MC. In contrast, NDP showed the strongest and weakest therapeutic effects in the non-CC/MC and CC/MC groups, respectively. Similar to NDP, CPT-11 showed a strong therapeutic effect in the non-CC/MC group, whilst the effect was not strong in those with CC/MC. For patients with CC, combination therapy of CPT-11 and cisplatin (CPT-P) have been used as an alternative to PTX and carboplatin (TC) therapy [22, 23]. However, a prospective randomized controlled trial failed to demonstrate the superiority of CPT-P therapy over TC therapy as a postoperative adjuvant therapy for CC [24]. This suggests that CPT-11 is not a particularly superior treatment for CC. Taken together, GEM might be recommended for patients with CC/MC, whilst CPT-11 and NDP might be recommended for those with non-CC/MC.

NDP has demonstrated limited efficacy for PR-OC. However, extending the platinum-free interval (using a nonplatinum-based regimen) might restore platinum sensitivity [25]. Patients with PR-OC who responded well to NDP had a longer PFI owing to treatment without platinum analogs after development of platinum resistance [26]. In our cohort, > 70% of NDP users received NDP after the third-line treatment after platinum resistance, indicating a sufficiently long platinum-free period post-platinum resistance diagnosis. Our findings demonstrated that a PFI ≥ 3 Mos could be a biomarker for an improved therapeutic effect of NDP against PR-OC (Fig. 3). Although platinum combination therapy is usually avoided in patients with PR-OC and a PFI < 6 Mos given the unfavorable relationship between intensity and efficacy, NDP might be an alternative treatment for patients with PFI ≥ 3 Mos after a sufficient platinum-free period.

Accumulating evidence suggests that Bev combination therapies significantly improve the PFS of patients with PR-OC [8–10]. In our study, Bev significantly improved TTF when combined with GEM and “others” (PTX and NGT), but not when combined with PLD, which is consistent with the JGOG3023 study [10] (Fig. 4). The background characteristics of our patients were more similar to those of the JGOG3023 study than the AURELIA study. Specifically, ~ 60% of patients had a PFI < 3 Mos and ~ 50% prevalence rate of SC, and there was no exclusion of third- or later-line treatments. Further large-scale cohort studies are warranted to validate the additive effect of Bev with PLD; however, it should be carefully considered given the increased risk of AEs.

The presence of ascites indicates a poor prognosis, regardless of the chemotherapy type. Preclinical studies on OC have shown that the presence of ascites promoted multidrug resistance in patients with OC [27, 28]. Our findings demonstrated that ascites was a hallmark of chemoresistance, regardless of chemotherapy type. Moreover, ascites volume positively correlated with the risk of AEs in CPT-11 users [29]; therefore, early CPT-11 use is recommended for PR-OC, especially for patients with non-CC/MC. Although the presence of ascites decreases the efficacy of chemotherapy agents, Bev can be used to control ascites [10]. Therefore, GEM + Bev or PTX + Bev might be better treatment options based on the compatibility of GEM and PTX with Bev.

Diarrhea and HFS/oral mucositis occurred only among CPT-11 and PLD users, respectively [5]. Additionally, PLD users showed the highest incidence of Grade ≥ 3 thromboembolic events (~ 10%). The incidence of thromboembolic events among PLD users varies among different studies [6, 7. The incidence of Grade ≥ 3 thromboembolic events in the AURELIA and REBECA trials among patients with PR-OC was ≤ 5% [8, 30]. In our study, the frequency of PLD-induced thromboembolic events surpassed that observed in these previous studies. Since our patients received more advanced- or late-line treatments than those in the AURELIA and REBECA trials, the high incidence of PLD-induced thromboembolic events might be partly attributed to cancer-related thrombosis accompanied by advanced cancer. Notably, PLD users showed the highest incidence of IP (3.8%), with most patients developing IP during the first two treatment cycles. Since thromboembolic events and IP could be life-threatening AEs, caution should be applied when administering PLD, especially in the first two treatment courses. Additionally, thromboembolic events occurred after the fourth treatment course, highlighting the need for continued vigilance during chemotherapy.

This study had a few limitations. First, we did not consider confounding factors when evaluating the efficacy of the drugs in terms of TTF. Therefore, our findings should be cautiously interpreted. Second, this was a single-center, retrospective, observational study. Accordingly, there may have been biases or tendencies in the selection of chemotherapy agents. For example, CPT-11 and NDP tended to be used in the first- and later-line treatments, respectively. The timing of drug use and selection of patients may have resulted in differences in treatment effects. Therefore, large-scale multi-center cohort studies are warranted to assess the heterogeneous effects of chemotherapy agents. Despite this, to the best of our knowledge, this is the largest single-center cohort study, which is based on data from 343 different treatments in 158 patients, making our findings more convincing. Third, some regimen cycles were longer than standard. These cycle duration extensions, paired with decreased dose intensity, may reduce chemotherapy efficacy. Consequently, interpreting our TTF findings requires caution. Furthermore, future research is needed to clarify the relationship between dosing interval and therapeutic effect.

In conclusion, we identified factors influencing the treatment efficacy of each chemotherapy agent used to treat patients with PR-OC. CPT-11/NDP and GEM were effective candidates for patients with non-CC/MC and CC/MC, respectively. NDP can be used as a late-line regimen for patients with PFI ≥ 3 Mos. Finally, PLD showed a relatively high incidence of thromboembolic events. Our findings could inform better selection of chemotherapy agents for PR-OC based on patient background and tumor characteristics.

## Supplementary Information

Below is the link to the electronic supplementary material.

Supplementary file1 (PPTX 337 KB)
