# Supplementary material for: Heterogeneous effects of cytotoxic chemotherapies for platinum-resistant ovarian cancer
Source: Int J Clin Oncol. 2023 Jun 22;28(9):1207–17. doi: 10.1007/s10147-023-02367-1 (PMC10468735; doi:10.1007/s10147-023-02367-1)
Supplement: Supplementary file 1 — Supplementary file1 (PPTX 337 KB) [file 10147_2023_2367_MOESM1_ESM.pptx]

## Slide 1
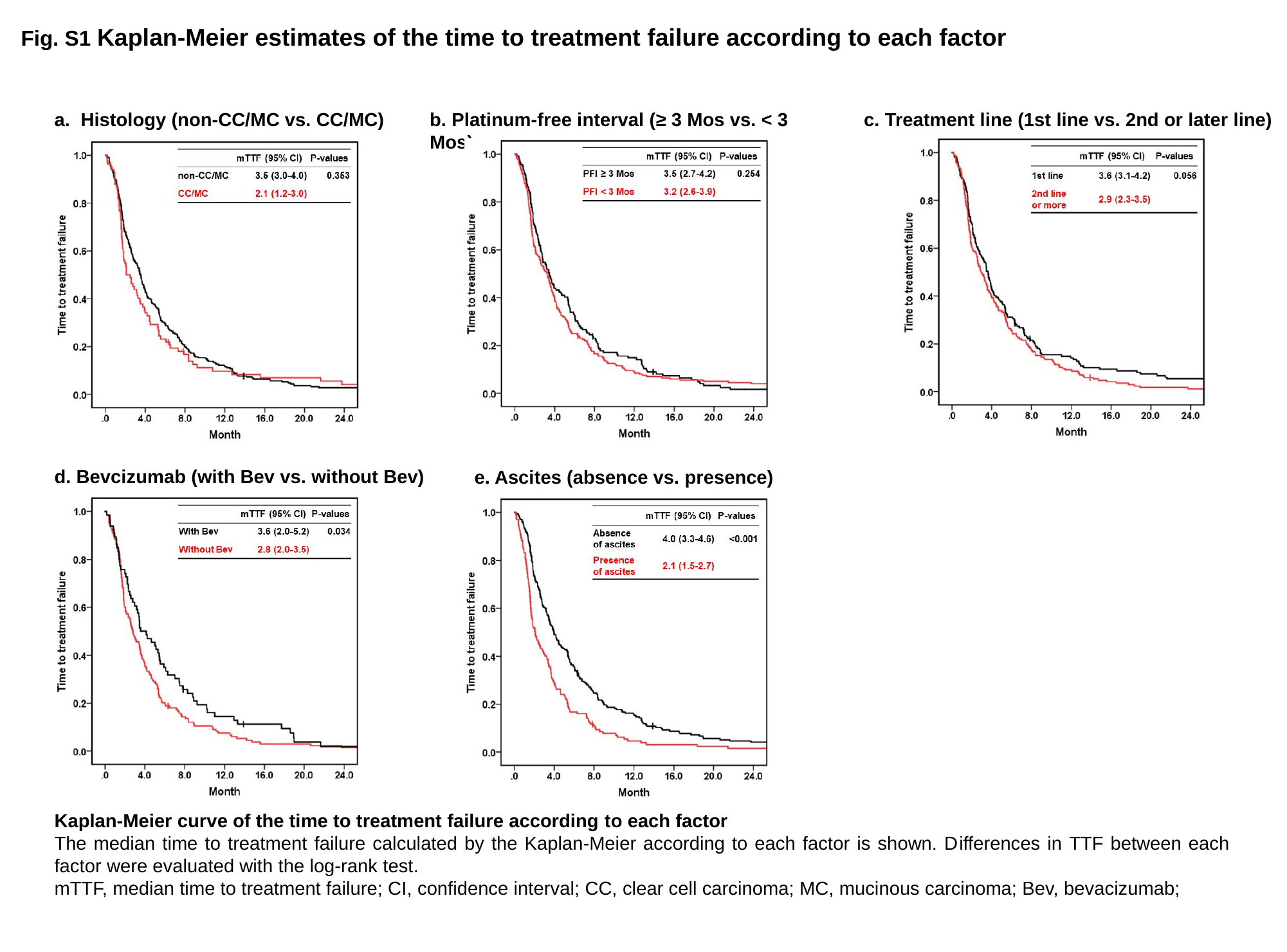

Fig. S1 Kaplan-Meier estimates of the time to treatment failure according to each factor
b. Platinum-free interval (≥ 3 Mos vs. < 3 Mos)
c. Treatment line (1st line vs. 2nd or later line)
a. Histology (non-CC/MC vs. CC/MC)
d. Bevcizumab (with Bev vs. without Bev)
e. Ascites (absence vs. presence)
Kaplan-Meier curve of the time to treatment failure according to each factor
The median time to treatment failure calculated by the Kaplan-Meier according to each factor is shown. Differences in TTF between each factor were evaluated with the log-rank test.
mTTF, median time to treatment failure; CI, confidence interval; CC, clear cell carcinoma; MC, mucinous carcinoma; Bev, bevacizumab;

## Slide 2
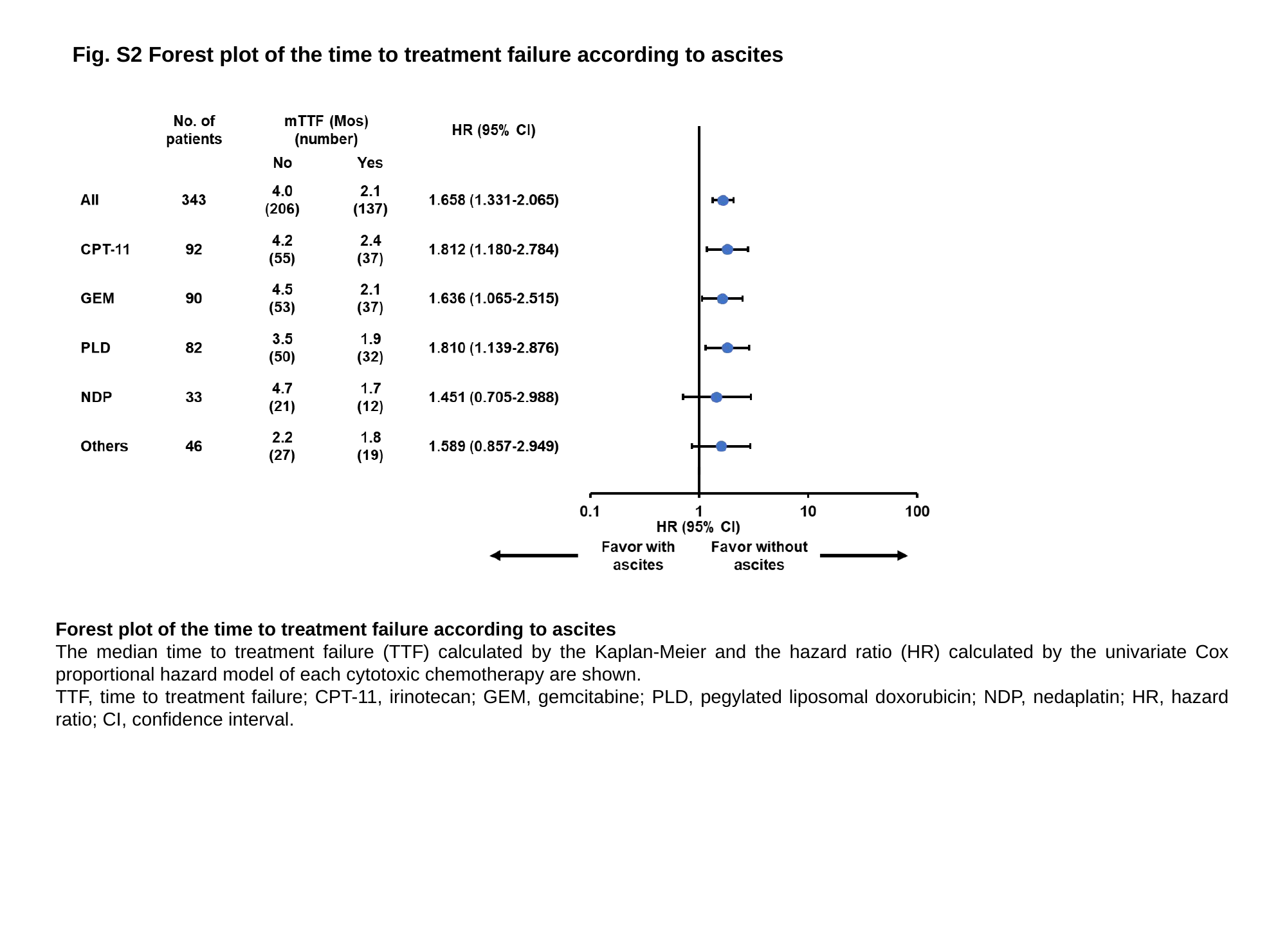

Fig. S2 Forest plot of the time to treatment failure according to ascites
Forest plot of the time to treatment failure according to ascites
The median time to treatment failure (TTF) calculated by the Kaplan-Meier and the hazard ratio (HR) calculated by the univariate Cox proportional hazard model of each cytotoxic chemotherapy are shown.
TTF, time to treatment failure; CPT-11, irinotecan; GEM, gemcitabine; PLD, pegylated liposomal doxorubicin; NDP, nedaplatin; HR, hazard ratio; CI, confidence interval.

## Slide 3
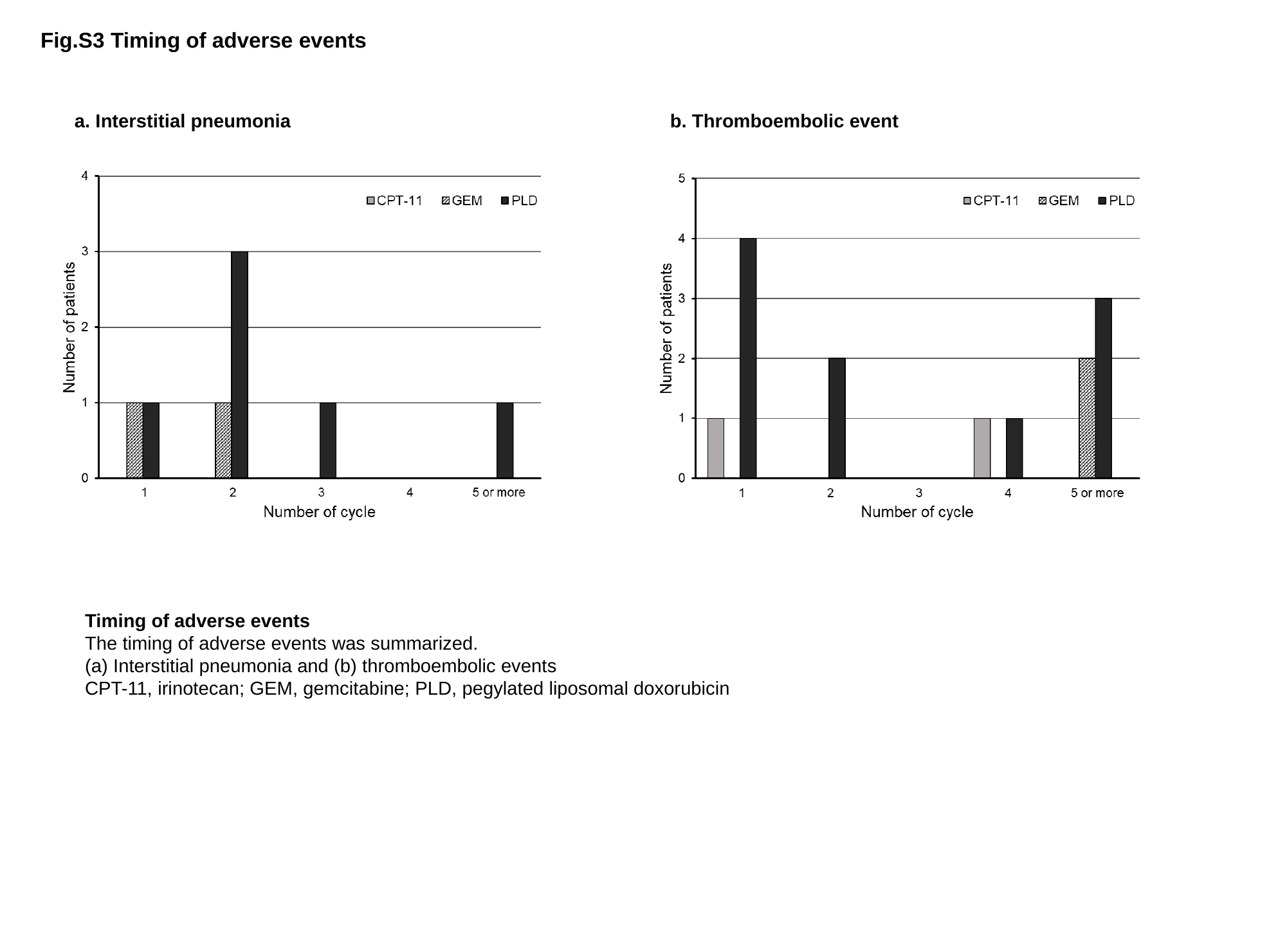

Fig.S3 Timing of adverse events
b. Thromboembolic event
a. Interstitial pneumonia
Timing of adverse events
The timing of adverse events was summarized.
(a) Interstitial pneumonia and (b) thromboembolic events
CPT-11, irinotecan; GEM, gemcitabine; PLD, pegylated liposomal doxorubicin
